# Modulation of monoaminergic neurotransmission in substance use disorders: a neuropharmacological perspective focusing on plant-derived metabolites

**DOI:** 10.3389/fphar.2026.1798820

**Published:** 2026-06-02

**Authors:** Bhagavathi Sundaram Sivamaruthi, Periyanaina Kesika, Chaiyavat Chaiyasut, Durairaj Ragu Varman

**Affiliations:** 1 Office of Research Administration, Chiang Mai University, Chiang Mai, Thailand; 2 Innovation Center for Holistic Health, Nutraceuticals, and Cosmeceuticals, Faculty of Pharmacy, Chiang Mai University, Chiang Mai, Thailand; 3 School of Biomedical Sciences, Sri Balaji Vidyapeeth (Deemed to be University), Puducherry, India

**Keywords:** essential oils, monoamine transporters, phytochemicals, polypharmacology, substance use disorders

## Abstract

Substance use disorders (SUDs) pose a major global health burden, often worsened by comorbid depression and anxiety. Dysregulation of monoaminergic neurotransmission, especially dopamine, serotonin, and norepinephrine transporters, underlies the reinforcing and harmful effects of addictive drugs. Current pharmacotherapies targeting these transporters offer benefits but are limited by delayed onset, side effects, and modest efficacy against emotional and cognitive symptoms. Despite advances in structural biology, a unified framework integrating transporter structure with docking and molecular dynamics simulations remains lacking. Emerging evidence suggests that plant-derived metabolites and essential oil preparations may modulate monoamine transporters through both direct and indirect mechanisms. These includes allosteric effects, membrane interactions, ion channel modulation, and downstream signaling pathways; however, these effects require further validation. This review summarizes recent preclinical and clinical data on transporter-modulating metabolites from *Hypericum perforatum* L., *Rhodiola rosea* L., *Withania somnifera*, and some of the essential oils. It highlights mechanistic insights from structural biology and molecular pharmacology, suggesting that plant-derived metabolites and essential oil preparations may influence monoaminergic neurotransmission and stress-related pathways. Despite challenges in bioavailability, standardization, and clinical validation, these metabolites may offer polypharmacology for adjunctive, personalized SUDs interventions. Integrated approaches, merging structural modeling, enhanced delivery, and rigorous trials, are needed.

## Graphical Abstract

**Figure:**
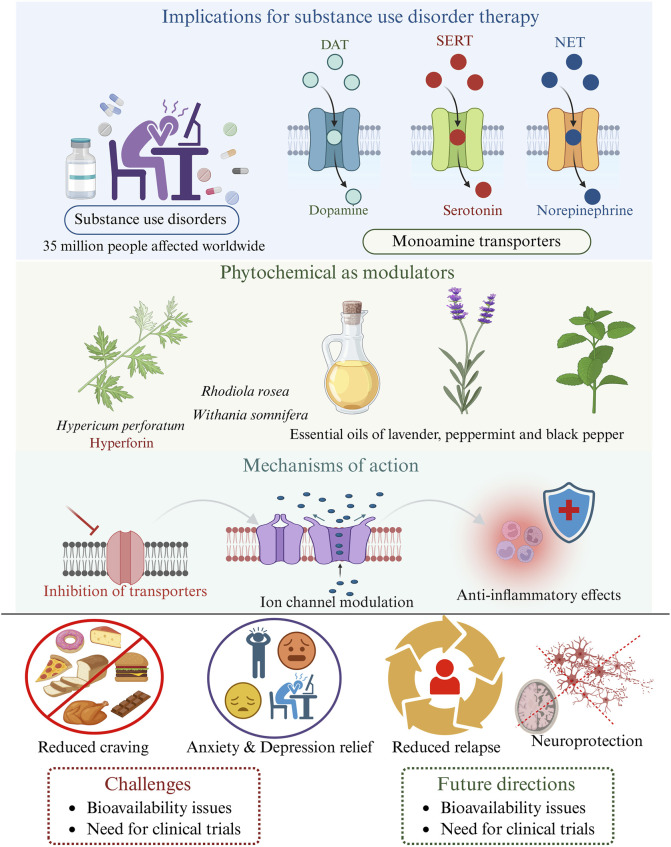
Schematic representation of monoamine transporter dysregulation in substance use disorders and its modulation by phytochemicals. Drugs of abuse alter dopamine (DAT), serotonin (SERT), and norepinephrine (NET) transporter function, leading to impaired reward processing, mood disturbances, and relapse vulnerability. Bioactive metabolites from medicinal plants and essential oils modulate transporter activity indirectly by regulating ion channels, altering membrane-associated mechanisms, and activating anti-inflammatory signaling. This balanced neuromodulation supports reduced craving, improved affective stability, lower relapse risk, and enhanced neuroprotection.

## 1 Introduction

Substance use disorders (SUDs) remain among the most urgent global health issues, affecting nearly 35 million people worldwide (Volkow et al., 2011). A key feature of addictive drugs is their ability to interfere with monoaminergic neurotransmission, mainly through interactions with dopamine (DAT), serotonin (SERT), and norepinephrine (NET) transporters. These plasma membrane proteins control extracellular monoamine levels by actively reabsorbing neurotransmitters from presynaptic terminals, thereby influencing synaptic signaling and neuroplasticity (Nestler, 2005).

Specific transporter systems have been linked to the reinforcing and harmful effects of various drugs of abuse.DAT is the primary target of cocaine and amphetamines, which block or reverse dopamine uptake to increase synaptic dopamine in the striatal reward pathways. Substances like 3,4-methylenedioxymethamphetamine and alcohol affect SERT, leading to serotonergic imbalance and mood disturbances. NET plays a crucial role in stress-induced relapse, as noradrenergic signaling contributes to arousal and cue-triggered craving (Volkow et al., 2011). Current pharmacotherapies targeting these transporters, such as selective serotonin reuptake inhibitors (SSRIs), serotonin–norepinephrine reuptake inhibitors (SNRIs), and bupropion, a dopamine and norepinephrine reuptake inhibitor, have consistently shown efficacy in randomized controlled trials by significantly increasing smoking abstinence rates, supporting a role for transporter modulation in nicotine dependence (Jorenby et al., 1999). SSRIs (sertraline and fluoxetine) have shown inconsistent efficacy in populations of alcohol and cocaine dependence (sometimes differentiated by subtypes of patients) or were not associated with significant reductions in SUDs (Carroll et al., 1994; Johnson et al., 2003). Atomoxetine was also tested in subjects with comorbid attention-deficit hyperactivity disorder and substance use disorder, resulting in improvements in cognitive symptoms, but with limited impact on addictive behaviors (Levin et al., 2009). Nevertheless, they have notable limitations, including adverse effects (sexual dysfunction, weight gain), delayed onset of action, high relapse rates, and reduced effectiveness in some patients (Vollenweider and Preller, 2020). These issues point to the need for safer, multi-target therapeutic strategies.

Pharmacological inhibition of these transporters is a cornerstone of antidepressant therapy. SSRIs and SNRIs act primarily by binding to and blocking these transporters, enhancing extracellular monoamine concentrations (Kuhar et al., 1991; Rudnick, 2006). Natural products present a promising avenue in this context. Plant metabolites have various pharmacological activities, such as modulation of monoamine transporters, antioxidant effects, neuroprotection, and regulation of stress responses (Sarris, 2007). Importantly, many of these metabolites act through polypharmacological mechanisms, simultaneously engaging multiple neurotransmitter systems and cellular pathways. This pleiotropy may be beneficial for treating SUDs, which involve complex neuroadaptations across dopaminergic, serotonergic, noradrenergic, and stress-related networks.

Although promising pharmacological results have been observed, key mechanistic gaps remain. Most studies on plant metabolites rely solely on functional assays and do not include structural transporter biology, *in silico* docking, or molecular dynamics simulations that could clarify conformational changes. This absence of computational and structural interaction analysis hampers rational drug optimization and translational advancement. This review highlights the importance of integrating plant metabolites and neuropharmacology with structural modeling and simulation-based transporter research. We emphasize structure–activity relationships, a mechanistic understanding of transporter modulation, and the therapeutic potential of these natural metabolites for addressing addiction and comorbid psychiatric disorders.

This narrative review was conducted by searching PubMed/MEDLINE, Scopus, and Web of Science, covering publications between January 2000 and March 2025. The search strategy combined keywords related to monoamine transporters (DAT, SERT, NET) with specific botanical terms and words related to addiction. Botanicals were selected based on either (i) direct experimental proof of affecting monoamine transporters or (ii) mechanistically supported indirect regulation of transporter-controlled monoaminergic neurotransmission in addiction-related contexts. Plants that function only as traditional high-potency monoamine oxidase inhibitors without documented transporter interaction were excluded from this review’s main focus.

Studies without taxonomic validation, standardized extracts, or thorough methodological details were excluded or interpreted with caution. Botanical nomenclature was verified using authoritative databases (World Flora Online and The Plant List). Due to heterogeneity in experimental models and outcome measures, quantitative meta-analysis was not performed. Methodological considerations such as extract characterization, pharmacological relevance, and experimental robustness were evaluated in accordance with best-practice recommendations for review articles. The review was evaluated in accordance with GA Best Practice recommendations for review articles, and the relevant checklist tables (1 and 2) are provided as Supplementary Material.

## 2 Plant metabolites as modulators of monoamine transporter function

### 2.1 Hypericum perforatum (st. John’s Wort)

*Hypericum perforatum* L. is among the most extensively investigated botanical drug preparations affecting monoamine systems. Most studies used hydroethanolic extracts of aerial parts standardized to hypericin (0.3%) and hyperforin (3% to 5%). Standardized extracts and their composition vary depending on whether they are extracted with ethanol or hydroethanol. Hyperforin, a lipophilic prenylated phloroglucinol derivative, broadly inhibits monoamine uptake. In synaptosomal assays, hyperforin inhibits the reuptake of serotonin, dopamine, and norepinephrine, with IC_50_ values typically near the low micromolar range (about 0.1 to 1 µM, depending on the assay) (Müller et al., 1998; Tian et al., 2014; Tu et al., 2010). Unlike typical competitive inhibitors such as fluoxetine, hyperforin does not directly bind to the S1 substrate site. Instead, it activates TRPC6 channels, which raise intracellular Na^+^ levels and disrupt the electrochemical gradient necessary for reuptake (Singer et al., 1999; El Hamdaoui et al., 2022).

In rodent studies, oral doses of 100 to 300 mg/kg of standardized extracts exhibit antidepressant-like effects in the forced swim and tail suspension tests. Positive controls in these studies usually include imipramine (10 to 20 mg/kg) and fluoxetine (10 mg/kg). During morphine withdrawal experiments, chronic treatment with the extract decreased hyperlocomotion and withdrawal-related anxiety behaviors, although relapse-specific endpoints are less studied. However, hyperforin is chemically unstable and induces CYP3A4, raising clinically relevant drug–drug interaction concerns. Besides hyperforin affecting transporter activity, *H. perforatum* contains hypericin and pseudohypericin, which can cause dose-dependent phototoxicity. Under exposure to UV or visible light, hypericin becomes photoactivated, leading to the formation of reactive oxygen species that may induce skin photosensitivity by damaging cells through oxidative processes. While serious phototoxic reactions are uncommon at typical antidepressant doses, they have been reported at higher extract levels or with prolonged use, particularly in individuals sensitive to light (Butterweck, 2003; Müller, 2003). These factors are important in long-term adjunct therapy. More importantly, hyperforin represents a potent inducer of cytochrome P450 3A4 (CYP3A4) through activation of the pregnane X receptor (PXR), resulting in increased transcription of drug-metabolizing enzymes (Izzo and Ernst, 2009; Markowitz et al., 2003).

In parallel, hyperforin-containing preparations upregulate p-glycoprotein (ABCB1), an ATP-dependent efflux transporter expressed in the intestinal epithelium and at the blood-brain barrier (BBB), thereby reducing systemic bioavailability and central nervous system penetration of co-administered agents. Significant decreases in plasma levels have been observed with various medications such as oral contraceptives, cyclosporine, tacrolimus, antiretrovirals, anticoagulants, benzodiazepines, and some antidepressants (Izzo and Ernst, 2009; Markowitz et al., 2003). Since individuals with substance use disorders often undergo polypharmacy, including antidepressants, opioid substitution therapy, or antiretroviral drugs, hyperforin’s induction potential presents a key translational obstacle. Consequently, diligent pharmacovigilance and the use of standardized extracts with controlled hyperforin levels are crucial when considering *H. perforatum* as an adjunct therapy. While transporter modulation is well-supported *in vitro*, direct transporter occupancy data in humans are lacking, and PET-based confirmation has not yet been established. Thus, translational interpretation must remain cautious.

### 2.2 Rhodiola rosea L.

*Rhodiola rosea L* (Crassulaceae), an adaptogenic plant traditionally used in Eastern Europe and Asia, has gained attention for its stress-protective and mood-enhancing effects. Although *R. rosea* does not significantly compete for binding at monoamine transporters, it affects monoaminergic tone by modulating enzymes and helping to normalize the stress response system. Since monoamine transporter function is closely associated with intracellular monoamine levels and stress-related neuroendocrine signaling, indirect modulation of monoamine activity could affect transporter-mediated neurotransmission in circuits relevant to relapse. Therefore, *R. rosea* is described as a neuromodulatory adaptogen rather than a traditional transporter inhibitor. Its primary bioactive metabolites include salidroside and rosavin. Pharmacologically, *R. rosea* extracts have been shown to inhibit monoamine oxidase A and B, thereby increasing monoamine levels and modulating serotonin and dopamine turnover (Ivanova Stojcheva and Quintela, 2022).

Studies primarily employed root extracts standardized to rosavins (3%) and salidroside (1%). Findings of monoamine oxidase (MAO)-A and MAO-B inhibition by standardized *R. rosea* extracts show effects at low to mid micromolar levels *in vitro* (Panossian and Wikman, 2010; Chaurasiya et al., 2022). Pharmacokinetic studies indicate that plasma concentrations of salidroside and rosavins after typical oral intake stay well below these IC_50_ thresholds (Hung et al., 2011; Ivanova Stojcheva and Quintela, 2022). Thus, their clinical effects are probably not due only to strong systemic MAO inhibition. Instead, they may involve adaptogenic and neuroendocrine mechanisms (Naoi et al., 2025), that influence stress response and cognitive function. In conditioned place preference (CPP) setups, *R. rosea* inhibits acquisition and expression of drug-conditioned reward and diminishes relapse behavior (Mattioli et al., 2012; Titomanlio et al., 2013), though long-term abstinence and relapse models need further exploration.

*In vivo* studies indicate that *R. rosea* influences monoaminergic activity and the hypothalamic-pituitary-adrenal (HPA) axis regulation (Panossian and Wikman, 2010; Ivanova Stojcheva and Quintela, 2022). However, there is no direct evidence of transporter occupancy or high-affinity binding at DAT, SERT, or NET in either animals or humans. Consequently, *R. rosea* likely acts mainly as a neuromodulatory adaptogen rather than as a high-affinity transporter inhibitor. Its therapeutic effects are likely due to stress buffering, neuroendocrine normalization, and indirect monoamine regulation, rather than direct reuptake inhibition.

Evidence suggests that salidroside may interact with transporter systems, although its effects appear less potent and more regulatory than hyperforin, which may be relevant for understanding potential therapeutic mechanisms in addiction-related contexts. The adaptogenic properties of *R. rosea*, enhancing resilience to physical and psychological stress, are relevant to addiction, as stress is a major trigger for relapse. Clinical studies indicate beneficial effects of *R. rosea* in stress-related fatigue, anxiety, and depression (Panossian and Wikman, 2010). Thus, while *R. rosea* may not act as a potent direct transporter inhibitor, its modulatory effects on monoamine dynamics and stress systems position it as a valuable adjunctive therapy for reducing relapse vulnerability in SUDs (Table 1). The results are encouraging, but the clinical evidence for *R. rosea* is limited by small sample sizes, short trial durations, and variations in extract composition and rosavin–salidroside ratios (Hung et al., 2011; Ivanova Stojcheva and Quintela, 2022). Many trials focus on stress-induced weariness instead of clearly defined mental health or drug use disorders, limiting their applicability to addiction populations. Moreover, direct evidence connecting *R. rosea* to monoamine transporter modulation in humans is predominantly inferential, as the majority of mechanistic data originates from *in vitro* experiments or rodent behavioral models (van Diermen et al., 2009; Mattioli et al., 2012). Thus, although adaptogenic and stress-buffering benefits are conceivable, comprehensive randomized controlled trials, particularly those assessing relapse prevention or desired outcomes in substance use disorder cohorts, remain insufficient.

**TABLE 1:** Evidence linking *Rhodiola rosea L.* to monoamine regulation, stress adaptation, and anti-addictive effects.

Study	Key findings	Relevance to monoamine transporters/addiction	References
*In vitro* MAO inhibition by *R. rosea*	Strong inhibition of MAO-A and MAO-B; rosiridin identified as an MAO-B inhibitor	Increases synaptic monoamines by reducing enzymatic breakdown	Van Diermen et al. (2009)
Adaptogens and CNS	*Rhodiola rosea* modulates the HPA axis, neurotransmitters, and stress response	Provides a mechanistic context for the neuroprotective/anti-addictive role	Panossian and Wikman (2010)
Stress and cognition	Adaptogens, including *Rhodiola rosea* improve stress-induced deficits	Suggests protective effects on cognitive function under drug stress	Llopis et al. (2025)
Chronic mild stress model	*Rhodiola rosea* extract reduced behavioural and physiological stress markers	Supports role in monoamine modulation under chronic stress	Mattioli et al. (2009)
Morphine CPP in mice	*Rhodiola rosea* blocked acquisition, expression, and reinstatement of morphine CPP	Demonstrates anti-addictive effect (opioids)	Mattioli et al. (2012)
Cocaine CPP in mice	Reduced acquisition/expression of cocaine CPP, but not reinstatement	Shows partial anti-addiction effect (psychostimulants)	Titomanlio et al. (2013)
Systematic review	Evidence supports efficacy in fatigue, stress, and mood	Clinical support, but limited direct addiction studies	Hung et al. (2011)

MAO: monoamine oxidase; CNS: central nervous system; HPA: Hypothalamic-Pituitary-Adrenal; CPP: conditioned place preference.

### 2.3 Withania somnifera

*Withania somnifera* (L.) Dunal (Solanaceae), commonly called ashwagandha, is one of the important plants in Ayurvedic medicine with broad adaptogenic, anxiolytic, and neuroprotective properties. Its pharmacological actions are primarily attributed to withanolides, a group of steroidal lactones. Investigations mainly used root extracts standardized to total *Withania somnifera* (2.5%-5%). Preclinical and clinical studies demonstrate that *W. somnifera* extracts normalize dopaminergic signaling, reduce cortisol levels, and exert robust anxiolytic and antidepressant-like effects (D'Cruz and Andrade, 2022; Dar et al., 2015; Singh et al., 2011). While direct binding to monoamine transporters has not been conclusively demonstrated, evidence suggests modulation of monoaminergic systems, possibly via indirect regulation of transporter expression and stress hormone pathways. In the context of addiction, Ashwagandha has been reported to alleviate withdrawal-induced anxiety and depressive symptoms in animal models, likely through its combined effects on dopamine, serotonin, and stress hormone regulation. These properties make *W. somnifera* a promising candidate for addressing both the affective and neurochemical dimensions of SUDs. Although there is growing clinical interest, direct mechanistic evidence linking *W. somnifera* to modulating monoamine transporters remains limited. Most research shows improvements in stress, anxiety, or cortisol levels instead of specific molecular effects on transporters (Speers et al., 2021; D'Cruz and Andrade, 2022; Arumugam et al., 2024). Preclinical results indicate dopaminergic regulation, but studies on direct binding, transporter occupancy, or conformational changes are rare. Furthermore, clinical trials tend to be short and mainly involve stressed or anxiety-prone individuals rather than those with substance use disorders. Therefore, while *W. somnifera* shows potential in reducing stress and improving mood, its precise role related to transporters in addiction remains speculative and warrants further targeted mechanistic and relapse-focused research.

It is important to emphasize that transporter modulation reports may vary substantially depending on extract preparation, concentration range, assay system, and membrane context. In several instances, monoamine transporter effects are observed only at micromolar concentrations that may exceed physiologically achievable plasma levels. Therefore, pharmacodynamic relevance must be interpreted alongside pharmacokinetic considerations, including bioavailability and BBB penetration.

## 3 Essential oils influencing monoamine transporter systems

### 3.1 *Lavandula angustifolia* (Lavender) *Oil*

Lavender oil is among the most extensively studied essential oils in neuropsychiatric research. Its major volatile metabolites, linalool and linalyl acetate, have been reported to exert both anxiolytic and antidepressant-like effects in animal models and clinical trials. Mechanistically, these metabolites interact with monoamine systems, though not always through direct transporter binding. Linalool, for example, enhances serotonergic neurotransmission and modulates glutamatergic and GABAergic activity, producing a net anxiolytic effect (dos Santos et al., 2022).

Silexan, a standardized oral pharmaceutical preparation of *Lavandula angustifolia* essential oil (typically standardized to defined concentrations of linalool and linalyl acetate), has been investigated in several clinical trials. Randomized controlled trials demonstrated its efficacy in reducing anxiety symptoms, with outcomes comparable to those of lorazepam and paroxetine but with a more favorable side-effect profile (Sivamaruthi et al., 2025; Kasper et al., 2010; Kasper et al., 2014; Dold et al., 2023; Woelk and Schläfke, 2010). Beyond its anxiolytic action, Silexan has also been shown to improve sleep quality and reduce somatic stress symptoms (Yap et al., 2019; Kasper and Eckert, 2025; Kasper et al., 2025).

Although lavender’s primary mechanism seems to involve modulation of serotonergic tone and GABA (gamma-aminobutyric acid) ergic activity, evidence for direct high-affinity interaction with SERT or DAT remains limited. Reported transporter-related effects are modest and probably reflect indirect modulation of transporter-regulated monoaminergic neurotransmission rather than typical competitive inhibition (Madonna et al., 2019; Dold et al., 2023; Kasper et al., 2018; Kasper et al., 2025). This indirect modulation makes lavender oil particularly useful as an adjunct therapy for managing comorbid anxiety in patients undergoing detoxification, where balancing efficacy with safety is critical. While silexan has shown effectiveness in randomized controlled trials for anxiety and mild-to-moderate depression, it is important to carefully examine some methodological concerns. Many studies focus on generalized anxiety disorder rather than substance use disorder populations, which limits their direct applicability to addiction settings (Kasper et al., 2010; Dold et al., 2023).

Additionally, essential oil formulations are prone to compositional fluctuation, and blindness may be partially undermined due to unique sensory characteristics, which can affect the reliability of studies assessing their therapeutic effects in anxiety and substance use disorder populations. Evidence supporting direct involvement of monoamine transporters is scarce; most research indicates that lavender oil indirectly influences serotonergic and GABAergic systems instead of strongly inhibiting transporters (dos Santos et al., 2022; Milanos et al., 2017). While lavender oil is typically well tolerated and effective at reducing anxiety, its potential to prevent relapse or serve in transporter-targeted addiction treatments requires further research via more detailed studies on substance use disorders. Therefore, lavender oil and Silexan should be considered neuromodulatory agents influencing monoaminergic systems indirectly, rather than primary monoamine transporter inhibitors.

### 3.2 *Mentha × piperita L.* (Lamiaceae) Oil

Peppermint oil, rich in menthol, has been widely studied for gastrointestinal and analgesic properties, but emerging evidence also links it to monoaminergic modulation. Menthol acts on transient receptor potential cation channel subfamily M (melastatin) member 8 (TRPM8) channels, indirectly influencing dopaminergic signaling in striatal regions. Recent animal studies have shown that menthol raises extracellular dopamine levels and boosts the dopaminergic effects of nicotine. This suggests that DAT modulation occurs in the nucleus accumbens and dorsal striatum (Umezu et al., 2021; Fan et al., 2016; Henderson et al., 2017). These findings are clinically relevant because nicotine addiction remains one of the most challenging forms of SUD, and menthol is known to alter smoking behavior.

While menthol’s role in facilitating nicotine reinforcement has raised public health concerns (Gardiner and Clark, 2010; van der Eijk et al., 2022; Rubin, 2024), at controlled doses, its regulatory effects on dopaminergic transmission may also be harnessed therapeutically. In preclinical models, low-dose menthol reduced cue-induced reinstatement of drug-seeking, suggesting a possible anti-craving effect (Nesil et al., 2019; Yahyavi-Firouz-Abadi and See, 2009; Marchant et al., 2013). Peppermint oil contains multiple bioactive constituents that may either increase or decrease addictive vulnerability depending on dose, context, and an individual neurochemical state. This duality requires dose-response studies and careful translational evaluation.

### 3.3 *Piper nigrum L.* (Piperaceae) *Oil*

Black pepper oil contains β-caryophyllene, a bicyclic sesquiterpene that has gained considerable interest in neuropsychopharmacology. Unlike many other essential oil metabolites, β-caryophyllene is a selective cannabinoid receptor 2 (CB2) receptor agonist that exerts strong anti-inflammatory and neuroprotective effects (Klauke et al., 2014). Importantly, it also demonstrates crosstalk with dopaminergic systems and has been shown to reduce nicotine self-administration and relapse in rodent models (He et al., 2020). The mechanism appears to involve CB2-mediated dampening of mesolimbic dopamine release, thereby reducing nicotine’s reinforcing effects. In addition, β-caryophyllene indirectly modulates monoamine transporter function via anti-inflammatory signaling, thereby normalizing dopamine–glutamate balance in addiction circuits (Ricardi et al., 2025; Mallmann and Oliveira, 2024).

Aromatherapy with black pepper essential oil has been reported to alleviate human cigarette cravings, possibly through sensory (oropharyngeal stimulation) and neurochemical mechanisms (Rose and Behm, 1994; Janes et al., 2020; Cortese et al., 2015). This dual-action sensory distraction and transporter-related modulation makes β-caryophyllene-containing oils a unique non-pharmacological intervention candidate for smoking cessation.

## 4 Mechanistic insights: transporter–ligand interactions

The DAT, SERT, and NET transporters are members of the solute carrier 6 (SLC6) family of secondary active transporters and are essential for maintaining synaptic monoamine homeostasis (Bu et al., 2021; Nepal et al., 2023). By coupling neurotransmitter reuptake to sodium and chloride gradients, these transporters ensure precise regulation of extracellular monoamine concentrations (Joseph et al., 2019; Nepal et al., 2023). The transporter’s function depends on conformational transitions between outward-open, occluded, and inward-open states throughout the transport cycle (Nguyen et al., 2024). High-resolution methods, including cryo-electron microscopy, have demonstrated that pharmacological inhibition is closely linked to the stabilization of conformational states, notably the outward-facing configuration that impedes neurotransmitter reuptake (Wu et al., 2025; Niello et al., 2020; Srivastava et al., 2024; Song and Wu, 2024; Su et al., 2025). Classical monoamine transporter inhibitors, such as selective serotonin reuptake inhibitors, cocaine, and bupropion, bind to the central substrate-binding pocket (S1 site) with high affinity (Aggarwal and Mortensen, 2023; Kristensen et al., 2011; Srivastava et al., 2024). Recent findings indicate that plant metabolites can affect transporter function via multiple pathways, such as orthosteric (S1) binding, allosteric (S2/vestibular) interactions, ion-gradient modulation, and membrane-dependent effects (Niello et al., 2020; Hasenhuetl et al., 2019; Chaurasiya et al., 2022; Nguyen et al., 2024; Naoi et al., 2025).

Canonical monoamine transporter inhibitors bind to the S1 site, stabilizing outward-facing conformations and raising extracellular monoamine levels. This mechanism explains the therapeutic effectiveness of SSRIs, SNRIs, and similar agents. However, prolonged or high-level transporter occupancy may lead to certain adverse effects in some individuals, such as emotional blunting or sexual dysfunction, depending on the dose, pharmacokinetics, and patient-specific factors (Cheng and Bhar, 2019; Agrawal et al., 2024; Aggarwal and Mortensen, 2023).

Conversely, several plant metabolites modulate monoamine transporter activity through indirect mechanisms, such as allosteric regulation, effects on membrane properties, and alterations in intracellular ionic balance (Schlessinger et al., 2023; Rebas et al., 2020; Kumar et al., 2025). A notable example is hyperforin, a prenylated phloroglucinol derivative isolated from *H. perforatum*. Rather than directly engaging with DAT, SERT, or NET, hyperforin activates TRPC6 channels, leading to increased intracellular sodium levels and a consequent reduction in the electrochemical gradient that drives transporter-mediated reuptake. This method induces broad, nonselective inhibition of monoamine transporters and represents one of the proposed mechanisms to simultaneously modulate DAT, SERT, and NET without involving the traditional substrate-binding site (Leuner et al., 2010; El Hamdaoui et al., 2022).

Comparable indirect mechanisms of action have been documented for terpenoids present in essential oils, such as linalool and β-caryophyllene. These metabolites typically exert neuromodulatory effects via membrane interactions and receptor-mediated signaling rather than by directly inhibiting transporters (Bellavite, 2023; Tkaczenko et al., 2025). Linalool enhances serotonergic neurotransmission by modulating presynaptic SERT activity and facilitates postsynaptic signaling via 5-HT_1_A receptors, resulting in anxiolytic and antidepressant-like effects in preclinical animal models (Milanos et al., 2017; dos Santos et al., 2022; Bavarsad et al., 2023).

β-Caryophyllene, a selective agonist of cannabinoid CB_2_ receptors, modulates dopaminergic function indirectly by diminishing stress-induced neuroinflammation and restoring transporter regulation, thereby supporting dopaminergic homeostasis and neuroprotection (Ricardi et al., 2025).

Collectively, these findings suggest that plant metabolites influence transporter activity rather than completely suppress. This may help preserving physiological neurotransmission and decreasing the risk of monoaminergic overstimulation. Analyses of structure-activity relationships clarify how the chemical characteristics of plant metabolites affect transporter regulation. Metabolites possessing lipophilic, prenylated, or terpenoid structures, such as hyperforin, adhyperforin, and caryophyllene, exhibit enhanced membrane permeability and demonstrate a preference for interacting with the hydrophobic regions of transporter proteins (Chaurasiya et al., 2022).

Computational docking and molecular dynamics analyses suggest that numerous plant metabolites stabilize transporter conformations through interactions with secondary allosteric vestibules, commonly referred to as S2 sites, rather than the primary S1 substrate-binding pocket (Beuming et al., 2008; Coleman et al., 2020; Hasenhuetl et al., 2019). Hyperforin and adhyperforin appear to incorporate into lipid bilayers and interact with transmembrane helices, thereby shifting the conformational equilibrium away from high-affinity substrate-binding states and reducing transporter efficiency (Jensen et al., 2001; El Hamdaoui et al., 2022).

Conversely, docking studies of smaller monoterpenes, such as linalool, reveal relatively low affinity for the SERT S1 site. Functional analyses, however, demonstrate considerable serotonergic modulation in relation to membrane composition and allosteric influences. This discrepancy highlights the limitations of docking approaches that omit lipid–protein interactions and emphasizes the necessity of membrane context in transporter modeling (dos Santos et al., 2022; Brown and Marrink, 2024). Menthol, a monoterpene, primarily modulates dopaminergic signaling by activating TRPM8 channels, thereby exerting an indirect influence on dopamine release. Initial computational data suggest marginal interactions with the vestibular areas of the DAT, requiring further experimental confirmation (Fan et al., 2016; Nguyen et al., 2024). These data collectively demonstrate that multi-site engagement and membrane-dependent mechanisms are characteristic of plant metabolites–transporter interactions, distinguishing them from the high-affinity, single-site binding typically observed with synthetic psychotropic medications.

A key advantage of plant metabolites is their inherent polypharmacology, enabling concurrent modulation of monoamine transporters, receptor systems, inflammatory pathways, and neuroplasticity mechanisms. This comprehensive pharmacological profile is particularly relevant in neuropsychiatric conditions such as substance use disorders, where dysregulation of monoamine transporters is associated with neuroinflammation, oxidative stress, HPA axis dysfunction, and impaired synaptic remodelling (Volkow et al., 2016; Volkow et al., 2011; Saggu et al., 2025). Hyperforin exhibits multimodal activity, combining significant monoamine transporter inhibition with anti-inflammatory effects mediated by peroxisome proliferator-activated receptor-γ activation (Müller, 2003). β-Caryophyllene integrates CB_2_ receptor activation with suppression of nuclear factor-κB signaling, thereby augmenting neuroprotection and stress resistance (Jha et al., 2021; Kalkan and Flammand, 2025). Similarly, withanolides derived from *W. somnifera* modulate monoaminergic signaling, regulate the HPA axis, and promote neurotrophic support and anti-inflammatory mechanisms rather than through demonstrated high-affinity binding to monoamine transporters (Mikulska et al., 2023; Pandit et al., 2024). This multi-target profile differs from highly selective transporter inhibitors, although both natural and synthetic metabolites may exhibit orthosteric or allosteric mechanisms depending on their molecular structure.

Despite these molecular advancements, many challenges remain that impede the clinical application of transporter modulation through plant metabolites. Bioavailability represents a significant concern, as hyperforin is chemically unstable and rapidly degrades, whereas volatile metabolites such as linalool and menthol exhibit limited penetration into the central nervous system (Patra et al., 2018; Di Francesco et al., 2023). Although indirect mechanisms of action may offer therapeutic advantages, they also raise concerns regarding off-target effects that can vary across different physiological contexts and individuals, potentially leading to unintended side effects or reduced efficacy in certain populations.

**FIGURE 1:**
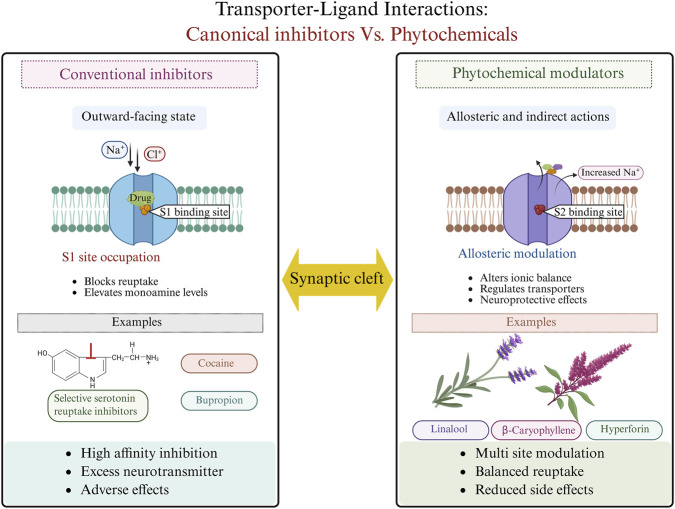
Interaction models between transporters and ligands: standard inhibitors versus phytochemical modulators. Traditional monoamine transporter inhibitors have a strong affinity for the core substrate-binding (S1) site. This keeps the outward-facing transporter conformations stable, but it also blocks much of the reuptake, which is undesirable. Phytochemicals may engage secondary vestibular (S2) sites, which function as allosteric modulatory regions, while others exert indirect effects through ion-channel activation or membrane-dependent conformational shifts. These tasks include working with secondary vestibular (S2) sites, activating ion channels, and altering membranes. This involvement at several sites may modulate transporter-associated monoaminergic signaling without complete competitive blockade while minimizing the risk of deleterious effects.

Furthermore, quantitative data on transporter binding kinetics for plant metabolites remain scarce, with most available evidence originating from preclinical or computational studies (Hasenhuetl et al., 2019). Genetic variation among individuals, especially polymorphisms in SERT and DAT, may influence the effectiveness of plant metabolites treatments, emphasizing the importance of personalized therapeutic strategies (Caspi et al., 2010; Hariri and Holmes, 2006). Subsequent investigations should prioritize integrative approaches that combine cryo-electron microscopy-resolved transporter structures with molecular docking, *in silico* ADMET modeling, and advanced nano-delivery systems to improve cerebral bioavailability and translational potential. These approaches may finally connect plant metabolites-neuropharmacology with clinically successful modulation of monoamine transporter function (Figure 1).

## 5 Therapeutic implications

The high prevalence of depression and anxiety among individuals with SUDs presents a major therapeutic challenge. Conventional pharmacotherapies largely focus on isolated neurotransmitter systems, whereas addiction involves coordinated neuroadaptations across multiple pathways (Volkow et al., 2016; Berríos-Cárcamo et al., 2020). This mechanistic complexity contributes to limited treatment efficacy and high relapse rates. In this context, metabolites of medicinal plants and essential oils may offer a potential advantage due to their intrinsic polypharmacology, enabling simultaneous modulation of neural circuits involved in affective, motivational, and stress-related processes. Extracts of *H. perforatum* influence serotonergic, dopaminergic, and noradrenergic signaling while attenuating withdrawal-associated behavioral and neurochemical alterations in preclinical models, with emerging evidence suggesting that hyperforin-mediated modulation of ion channels and downstream neuroimmune effects contribute to its broader therapeutic profile (Butterweck, 2003; Müller, 2003).

Adaptogenic metabolites such as *R. rosea* enhance stress resilience and normalize HPA axis dysregulation, mechanisms increasingly recognized as central to stress-induced relapse and negative affective states in addiction (Ivanova Stojcheva and Quintela, 2022). Similarly, β-caryophyllene, a selective cannabinoid CB2 receptor agonist, reduces nicotine self-administration and drug-seeking behaviors while exerting anti-inflammatory and neuroprotective actions, supporting a functional link between immune modulation and addiction-related neuroplasticity (He et al., 2020; Ricardi et al., 2024; Ricardi et al., 2025).

Beyond monotherapy, plant metabolites may serve as potential adjuncts to standard pharmacological interventions. Lavender oil preparations such as Silexan have demonstrated anxiolytic efficacy comparable to benzodiazepines but with superior tolerability and minimal dependence liability, suggesting utility during detoxification or early abstinence when anxiety is pronounced (Kasper et al., 2010). *Withania somnifera* has shown clinically relevant stress-reducing and mood-stabilizing effects and may improve treatment adherence when combined with antidepressants, particularly in patients with high stress reactivity (Speers et al., 2021; Arumugam et al., 2024). Although menthol-containing peppermint oil requires cautious dosing due to potential reinforcing properties, controlled exposure has been reported to attenuate craving and cue-induced responses, indicating context-dependent therapeutic potential (Harrison et al., 2017; Johnson et al., 2022; Bello et al., 2024). Despite these promising effects, several translational barriers persist, including variability in composition, limited bioavailability and central nervous system penetration, insufficient large-scale clinical trials specifically targeting the people with SUDs, and the risk of clinically relevant botanical drug interactions. Preparations of *H. perforatum* that contain hyperforin activate the PXR. This activation increases the expression of CYP3A4 and raises p-glycoprotein levels, which enhances drug metabolism and efflux. Consequently, it can reduce the plasma and central nervous system concentrations of co-administered drugs such as oral contraceptives, immunosuppressants, antiretrovirals, anticoagulants, benzodiazepines, and some antidepressants (Izzo and Ernst, 2009; Sarris, 2018; Markowitz et al., 2003). Patients with substance use disorders often receive multiple medications, such as opioid substitution therapy and psychotropic drugs. Careful monitoring and standardized extract selection are crucial when considering *H. perforatum* as an adjunct treatment. Addressing these limitations through standardized formulations, advanced delivery strategies, and rigorous clinical validation will be essential for integrating plant metabolites into evidence-based pharmacotherapy for addiction and its psychiatric comorbidities.

Moving forward, plant metabolites are unlikely to replace synthetic transporter inhibitors but could serve as complementary therapies. A future treatment landscape may move toward personalized medicine, where individuals with genetic polymorphisms in transporter genes, such as 5-HTTLPR within SERT, or those with heightened relapse vulnerability, receive tailored plant metabolite–based adjunct therapies aligned with their neurobiological profile.

Such approaches would align with holistic management of SUDs: addressing neurochemical imbalances, alleviating psychiatric comorbidities, and enhancing treatment adherence through better tolerability and reduced side effects (Table 2). Importantly, the activity of multi-target plant metabolites should not be assumed to be inherently safer than conventional transporter inhibition. Some botanical constituents exhibit clinically relevant pharmacokinetic interactions, including CYP3A4 and p-glycoprotein induction (e.g., hyperforin) or inhibition (e.g., piperine), highlighting the need for careful pharmacovigilance.

**TABLE 2:** The representative bioactive plant-metabolites, possible mechanism of action and their therapeutic relevance.

Plant materials	Active metabolites	Transporter targets	Mechanism of action	Preclinical/Clinical evidence	Therapeutic relevance	References
*Hypericum perforatum L.*	Hyperforin, adhyperforin, hypericin	SERT, DAT, NET	TRPC6 activation → Na^+^ gradient modulation → broad reuptake inhibition	Preclinical: ↓ morphine withdrawal, ↓ ethanol intake. Clinical: effective antidepressant	Depression + withdrawal symptoms	Müller (2003), Butterweck (2003)
*Rhodiola rosea L.*	Salidroside, rosavin	Indirect modulation of SERT, DAT, NET	MAO-A/B inhibition, ↑ 5-HT, and DA turnover	Clinical: stress, fatigue, mild depression	Stress resilience, relapse prevention	Panossian and Wikman (2010)
*Withania somnifera* (L.) dunal	Withanolides	Indirect dopaminergic/serotonergic modulation	↓ Cortisol, normalization of monoamine signalling	Preclinical: anxiolytic, antidepressant. Clinical: reduces stress/anxiety	Withdrawal anxiety, comorbid depression	Singh et al. (2011), Mikulska et al. (2023)
*Lavandula angustifolia*	Linalool, linalyl acetate	SERT (indirect), GABA-A	Enhances serotonergic/GABAergic tone	Clinical: effective in generalized anxiety disorder	Anxiety management during detox	Kasper et al. (2014), Kasper et al. (2010)
*Mentha × piperita L*	Menthol	DAT (indirect via TRPM8)	↑ DA release modulates nicotine reward	Preclinical: ↑ striatal DA modulates nicotine reinforcement	Craving regulation, smoking cessation	Fan et al. (2016)
*Piper nigrum L*	β-Caryophyllene	DAT (indirect), CB2 receptor	CB2 agonism, ↓ mesolimbic DA release	Preclinical: ↓ nicotine self-administration. Clinical: ↓ cigarette craving	Smoking cessation, relapse prevention	He et al. (2020), Rose and Behm (1994)

SERT: serotonin transporter; DAT: dopamine transporter; NET: norepinephrine transporter; GABA-A: Gamma-aminobutyric acid type A; CB2: Cannabinoid receptor 2; TRPM8: Transient receptor potential cation channel subfamily M member 8; MAO: monoamine oxidase; 5-HT: 5-hydroxytryptamine; DA: dopamine.

## 6 Limitations and safety considerations

Despite their promising neuromodulatory properties, these plant metabolites raise critical considerations regarding safety and successful translation into clinical practice. Hyperforin-containing preparations of *H. perforatum* activate the PXR, which increases CYP3A4 activity and upregulates p-glycoprotein. This may reduce the plasma and central nervous system levels of co-administered drugs, such as oral contraceptives, immunosuppressants, antiretrovirals, anticoagulants, antidepressants, and opioid substitution therapies. Hypericin-related phototoxicity has also been observed at higher doses or with prolonged exposure. Essential oils warrant comparable caution, as menthol may, under certain conditions, potentiate nicotine reinforcement, reflecting its dose- and context-dependent dual actions. Although *W. somnifera* and *R. rosea* are generally well tolerated, most clinical trials are short-term and frequently exclude polypharmacy populations typical of substance use disorder settings, leaving long-term safety in these groups insufficiently characterized. Pharmacokinetic and formulation-related constraints further complicate translation.

Many plant metabolites encounter challenges such as limited oral bioavailability, chemical instability, or difficulty crossing the BBB. In several experimental models, transporter modulation happens at micromolar concentrations, which are often higher than normal plasma levels. Additionally, variations in extract composition, such as different levels of hyperforin, rosavin-salidroside ratios, or essential oil terpene profiles, can lead to inconsistent pharmacological effects, further emphasizing the necessity of precise standardization. Due to differences in experimental designs, outcome measures, and extract preparations, quantitative synthesis was not possible. Additionally, most mechanistic evidence comes from *in vitro* assays, animal models, or computational simulations, with limited human transporter occupancy data and few relapse-focused randomized controlled trials in people with substance use disorder. Therefore, although mechanistic plausibility is supported, the clinical relevance remains preliminary and should be approached cautiously.

## 7 Future directions

Although evidence supports the potential of plant metabolites and essential oils to modulate monoamine transporters, a major translational limitation remains the insufficient integration of structural biology to validate plant metabolite-transporter interactions. Recent breakthroughs in cryo-electron microscopy have revealed transporter conformations at near-atomic resolution (Coleman and Gouaux, 2018). This structural knowledge allows computational docking and molecular dynamics simulations to predict how plant metabolites interact with transporters in distinct conformational states. For example, docking of hyperforin analogues into human SERT suggests binding within the allosteric S2 vestibule rather than the canonical S1 site (Plenge et al., 2020; Zhu et al., 2020).

These techniques can identify crucial structural features such as prenylation, terpenoid backbones, and hydroxyl groups that influence binding affinity. Such an approach supports the systematic creation of enhanced synthetic-natural hybrid molecules. However, the main challenges in applying these metabolites are their poor bioavailability and stability, which hinder their practical use. Hyperforin is chemically unstable, and volatile metabolites like linalool and menthol do not readily cross the BBB. Nanotechnology-based delivery systems, like liposomes, solid lipid nanoparticles, and polymeric nanocarriers, may improve solubility, protect against degradation, and aid in distributing metabolites within the central nervous system (Patra et al., 2018; Lu et al., 2021; Egba et al., 2025). Nanoencapsulation has already increased the effectiveness of linalool in preclinical studies and may also boost the *in vivo* efficacy of β-caryophyllene and withanolides (Shi et al., 2016; An et al., 2021).

Translation is significantly more challenging because individuals differ greatly. Many findings related to monoamine transporters derive from *in vitro* uptake assays or acute rodent behavioral paradigms, which do not fully capture relapse vulnerability, compulsive drug-seeking behavior, or the neurobiological complexity of human addiction (Marchant et al., 2013; Nestler, 2005; Volkow et al., 2016). While *in silico* docking and molecular dynamics studies provide important structural insights into ligand–transporter interactions (Beuming et al., 2008; Plenge et al., 2020; Nguyen et al., 2024), these approaches cannot substitute for experimental validation in membrane-embedded transporter systems, electrophysiological assays, or *in vivo* occupancy studies (Hasenhuetl et al., 2019; Niello et al., 2020). Moreover, several plant metabolites’ effects are observed only at micromolar concentrations in biochemical assays, levels that may exceed physiologically achievable plasma concentrations following oral administration (Patra et al., 2018; Di Francesco et al., 2023). Future research should incorporate validated relapse–reinstatement models and chronic administration paradigms (Marchant et al., 2013). It should also include transporter occupancy measurements, pharmacokinetic–pharmacodynamic integration, and clinically relevant endpoints such as craving intensity and long-term abstinence (Volkow et al., 2016; Saggu et al., 2025). Polymorphisms in transporter genes such as SLC6A3 and SLC6A4 influence individual responses to both synthetic and natural modulators (Zhu et al., 2017; Miozzo et al., 2020; Lachowicz et al., 2025). Genetic stratification, along with pharmacogenomic and metabolomic testing, could aid in identifying patient subgroups most likely to benefit from additional plant metabolite-based adjuvant therapy. Although preclinical results are promising, clinical data remain limited. Controlled trials show lavender oil’s effectiveness for anxiety and *H. perforatum* for depression. However, there is a lack of extensive randomized studies focusing on addiction-related outcomes, such as cravings, relapse prevention, and withdrawal. Future trials should utilize standardized, chemically characterized extracts to improve reproducibility, implement adjunctive designs that integrate plant metabolites with SSRIs, SNRIs, or behavioral interventions, and include longitudinal relapse-prevention outcomes instead of concentrating exclusively on acute withdrawal or short-term mood assessments. From a regulatory perspective, plant-derived bioactives may be classified either as botanical drugs (requiring FDA/EMA approval pathways, as exemplified by Silexan) or as nutraceutical adjuncts. The former ensures pharmacological rigour, while the latter increases accessibility but risks inconsistency.

## 8 Conclusion

Some of the metabolites, including essential oil, act as distinctive modulators of monoamine transporter systems. Unlike conventional high-affinity inhibitors that directly compete at binding sites, many of these metabolites influence transporter activity through allosteric, membrane, or ion-channel mechanisms, offering a more balanced regulation of monoamines. This pharmacological profile closely matches the complex neurobiological features of substance use disorders, including transporter dysfunction, neuroinflammation, stress-axis imbalance, and maladaptive neuroplasticity. Although some plant metabolites have clinical support for depression and anxiety, their use in addiction treatment is still mainly in the preclinical research phase. Thus, plant metabolites should be seen as promising complementary options rather than standalone solutions for SUDs. Future advancements will rely on creating standardized formulations, improving bioavailability, applying pharmacogenomic stratification, and conducting thorough clinical trials focused on preventing relapse and reducing cravings. With proper translational validation, plant metabolite-based transporter modulators may contribute to integrated, multi-target strategies for addiction. However, their clinical application requires careful evaluation of safety, drug interactions, and extract standardization.
