# Supplementary material for: Modulation of monoaminergic neurotransmission in substance use disorders: a neuropharmacological perspective focusing on plant-derived metabolites
Source: Front Pharmacol. 2026 Jun 2;17:1798820. doi: 10.3389/fphar.2026.1798820 (PMC13269353; doi:10.3389/fphar.2026.1798820)
Supplement: Supplementary file 1 [file Table1.docx]

**Supplementary Material: GA Best Practice Tables 1 and 2**

**Supplementary Table 1. GA Best Practice – Review Methodology Compliance**

| **S.No** | **Criterion** | **Compliance Statement** |
| --- | --- | --- |
| 1 | Type of article | Narrative review with structured literature search |
| 2 | Databases searched | PubMed/MEDLINE, Scopus, Web of Science |
| 3 | Time frame | January 2000 – March 2025 |
| 4 | Inclusion criteria | Peer-reviewed in vitro, in vivo, and clinical studies evaluating phytochemical modulation of monoamine transporters |
| 5 | Exclusion criteria | Studies lacking methodological transparency, taxonomic clarity, or pharmacological relevance |
| 6 | Botanical nomenclature considered | Yes – critically evaluated where reported |
| 7 | Extract characterization assessed | Yes – evaluated against ConPhyMP principles |
| 8 | Chemical fingerprinting assessed | Yes – methods (TLC, HPLC, LC-MS, GC-MS, NMR) evaluated when reported |
| 9 | Marker compound quantification evaluated | Yes |
| 10 | Reproducibility and batch consistency assessed | Yes |
| 11 | Limitations and methodological gaps discussed | Yes |
| 12 | Quantitative synthesis | Not performed due to heterogeneity |

**Supplementary Table 2. Scientific Quality and Extract Characterization Appraisal (ConPhyMP-Aligned)**

| **Domain** | **Assessment Performed** | **Summary** |
| --- | --- | --- |
| Botanical authentication | Evaluated | Reported inconsistently; discussed as a limitation |
| Voucher specimen deposition | Evaluated | Frequently absent; identified as reporting gap |
| Extract standardization (DER/DSR) | Evaluated | Often incomplete; variability discussed |
| Extraction solvent and parameters | Evaluated | Not uniformly reported across studies |
| Chemical fingerprinting | Evaluated | HPLC and LC-MS are common; multi-orthogonal fingerprinting is rare |
| Marker compound quantification | Evaluated | Present in some studies; justification often limited |
| Pharmacopoeial compliance | Evaluated when applicable | Inconsistent across studies |
| Reference standards used | Evaluated | Variable reporting |
| Batch reproducibility | Evaluated | Rarely reported |
| Translational validity | Critically assessed | PK/PD integration is limited |
| Overall strength of evidence | Moderate (preclinical), limited (clinical SUD) | Major need for standardized, relapse-focused RCTs |
